# Early Life Trauma Has Lifelong Consequences for Sleep And Behavior

**DOI:** 10.1038/s41598-019-53241-y

**Published:** 2019-11-13

**Authors:** Monica Lewin, Jenna Lopachin, James Delorme, Maya Opendak, Regina M. Sullivan, Donald A. Wilson

**Affiliations:** 1 Emotional Brain Institute, Nathan Kline Institute for Psychiatric Research, Orangeburg, NY USA; 2 Sackler Neuroscience Graduate Program, NYU School of Medicine, New York, NY USA; 3 Department of Child and Adolescent Psychiatry, NYU School of Medicine, New York, NY USA; 4 Neuroscience Graduate Program, University of Michigan, Ann Arbor, MI USA

**Keywords:** Stress and resilience, Sleep deprivation

## Abstract

Sleep quality varies widely across individuals, especially during normal aging, with impaired sleep contributing to deficits in cognition and emotional regulation. Sleep can also be impacted by a variety of adverse events, including childhood adversity. Here we examined how early life adverse events impacted later life sleep structure and physiology using an animal model to test the relationship between early life adversity and sleep quality across the life span. Rat pups were exposed to an Adversity-Scarcity model from postnatal day 8–12, where insufficient bedding for nest building induces maternal maltreatment of pups. Polysomnography and sleep physiology were assessed in weaning, early adult and older adults. Early life adversity induced age-dependent disruptions in sleep and behavior, including lifelong spindle decreases and later life NREM sleep fragmentation. Given the importance of sleep in cognitive and emotional functions, these results highlight an important factor driving variation in sleep, cognition and emotion throughout the lifespan that suggest age-appropriate and trauma informed treatment of sleep problems.

## Introduction

Sleep quality varies widely across healthy individuals, especially in normal aging, and reduced quality of sleep can disrupt cognitive and emotional functioning, decreasing quality of life and mental health^1^. Ample clinical work has shown that sleep disturbances are also a strong predictor of subsequent psychiatric diagnosis^2,3^, and insomnia is the most ubiquitous symptom shared across the range of disorders in the DSM^4,5^. However, while the link between early life adversity and various psychiatric disorders is well established^2–6^, it is only recently that the link between early life adversity and sleep quality has received clinical attention^6–8^. The goal of the present work is to further assess the link between early life adversity and sleep using an animal model of early life maltreatment from the mother and assessment of subsequent sleep quality across the lifespan.

We take a lifespan approach to assess the link between early life adversity and sleep because the structure of sleep changes through development and adulthood in humans^9,10^. For example in children, slow wave activity (SWA) spectral power during non-rapid eye movement (NREM) sleep decreases with cortical maturation during development^11–13^. Furthermore, many aspects of sleep physiology and sleep architecture appear to be altered in different psychopathological states, and across the lifespan^6^. Decreases in NREM sleep spindles and alterations in NREM spectral power have been linked to various emotional and behavioral problems in children and adolescents^14,15^, as well as to dementia^16^ in later life. Clinical depression, also a well-known consequence of developmental trauma^17–22^, has been shown to present with reductions in slow wave sleep, NREM sleep fragmentation, and increases in rapid eye movement (REM) sleep parameters^23^. Unfortunately, research that characterizes human sleep across development remains scarce^9^. The development of sleep has also been explored in rodents, but the effects of early life adversity on sleep primarily focus on adult outcomes^8,24,25^. Here, we ask how early life adversity outcomes across the lifespan interact with the changes in sleep architecture which occur across development.

Early life adversity, especially abusive caregiving, is known to produce lifelong changes in neurobehavioral functioning that dramatically increases the risk of medical and public health problems, including addiction, crime, health and psychiatric illness^26^, in both humans and animal models^17–22,27,28^. a history of developmental trauma is also associated with a greater incidence of sleep problems^29–31^ and recent clinical evidence suggests that normalizing sleep may have global benefits for psychiatric patients^32,33^. Thus, identifying sleep targets for therapeutic intervention is an important translational consideration for survivors of early life trauma. To this end, we used a Scarcity-Adversity animal model of early life maltreatment, which produces a neurobehavioral depressive-like outcome^19–21^.

Our animal model of early life adversity is the Scarcity-Adversity model of Low Shavings, where the mother is provided with insufficient bedding to construct a nest while pups are postnatal (PN) days 8–12. This procedure stresses the mother and induces maternal maltreatment of pups. Following this brief intervention, the dam and litter were again provided normal bedding levels until testing, which restores typical maternal behavior. Ages for testing included newly independent (weanlings) rats, young adulthood, and older adulthood to provide a life span approach. We assessed multiple features of sleep architecture, microstructure, and sleep fragmentation as well as behavioral activity levels in and outside the home cage. The results demonstrate that adult sleep quality can be shaped by early life adverse events presenting an age-specific dynamic effect. Given the importance of sleep in cognitive and emotional functions, these results highlight an important factor driving variation in sleep and cognition throughout the lifespan that suggest age-appropriate and trauma informed treatment of sleep problems.

## Methods

### Subjects

A total of 135 Long Evans hooded rats, 75 males and 60 females, bred and reared in our animal facility and maintained on *ad lib* food and water. Litters were culled on PN1 (day of birth is PN0) to 12 pups per litter (~ equal numbers of male and female), cages were cleaned twice a week, although the nest was retained and placed into the cleaned cage. Animals were selected from a total of (41 used for sleep and activity + 18 used in open field) different litters (33 sleep + 27 open field) females and (48 + 27) males were used. All handling, housing and experimental procedures were approved by, and performed in accordance with, both the Animal Care and Use Committee and NIH guidelines.

### Scarcity-adversity model of maltreatment

#### Low bedding (LS) rearing manipulation

Early-life trauma was induced using a Scarcity-Adversity Model of low bedding from postnatal days (PN)8–12, where insufficient bedding for nest building induces maternal maltreatment of pups^34–37^. This rearing manipulation (“LS” or low wood shavings bedding condition) involves the removal of all but 100 ml of the bedding material (a thin layer covering half the floor remains). The bedding is replaced daily, to maintain a clean cage. Comparison control litters (CON condition) were reared at the same time and treated similarly in all respects, except sufficient bedding is provided (4500 ml). This procedure is validated to produce abusive behavior in the mother (i.e. stepping on pups, dragging pups) and results in limbic system dysfunction and abnormal depressive-like behavior and abnormal responses to threat, though pups grow and gain weight normally^35^. We target this treatment to PN8–12 in the rodent pup; though there is no consensus aligning rodent age onto human age, this developmental period corresponds roughly to toddlerhood^38^. In the rodent, this is a sensitive period for attachment learning and a period of neurobehavioral transitions including increased amygdala engagement in threat processing. This major developmental transition is also associated with pups’ initial decrease on full reliance on the mother: pups begin nibbling on solid food and occasionally take brief excursions out of the nest^39^. Targeting the LS treatment to this developmental period has been shown to produce robust immediate outcomes on neurobehavioral function at PN13 revealed by increasing stress^40^, and fully emerge at weaning with altered dynamic, developmental trajectories through adolescence and adulthood^20,41^.

In a subset of animals, daily videos were collected during the 5 day LS procedure to validate changes in maternal care. Videos were scored for maternal behaviors using BORIS software by two independent raters. Hour-long videos during the first half of the light phase from control and LS litters were segmented into 5 minute bins and the percent of bins where given behaviors were observed was calculated (see Table 1). Inter-rater correlation was 0.97.

**Table 1:** Scarcity-Adversity model of low shavings (LS) induces abusive maternal behavior to pups for 5 days beginning when pups are postnatal day (PN)8.

Maternal Behavior	Control (% of obs)	LS (% of obs)	Stat
Nursing	52.78 ± 19.44	50 ± 9.62	*t*_(4)_ = 0.13, *p* = 0.9
In nest	75 ± 14.43	80.56 ± 12.11	*t*_(4)_ = 0.29, *p* = 0.78
Step on pups	**11.11 **±** 2.78**	**52.78 **± 2.78*	*t*_(4)_ = 10.6, *p* < 0.001
Scattered litter	**2.78 **±** 2.78**	**50 **±** 17.34**^**#**^	*t*_(4)_ = 2.69, *p* = 0.054
Drag pups	**0 **±** 0**	**27.78 **± 2.78*	*t*_(4)_ = 10, *p* < 0.001
Average pup weight (g)	24.07 ± 0.45	22.42 ± 0.69	*t*_(4)_ = 1.54, *p* = 0.19

Rat mothers have a strong motivation to build a nest for the pups and providing insufficient material (wood shavings) stresses the mother and induces rough handling of pups, such as stepping on pups, dragging pups out of the nest and scattering the litter. The rough handling does not produce any visible signs on pups. Nurturing maternal behaviors such as time in the nest and nursing occur at similar levels to controls and LS pups gain weight and appear to mature at a level similar to controls pups. N’s = 3 pups/group, 1 pup/litter.

### Telemetry recordings

LS and control rats were implanted with wireless telemetry packs (F20-EET, DSI Inc) shortly after weaning from the mother at PN21 (PN 21–25, N = 25), at young adulthood (3–5 months, N = 20), or at older adulthood (7–16 months, N = 36). For all ages, rats were anaesthetized with isoflurane anesthesia and a custom low-impedance local field potential (LFP) recording electrode attached to the telemetry lead (100 µm diameter stainless steel) was placed in the left frontal cortex (adult approximate coordinates: anteroposterior [AP]: +4.5 mm, mediolateral [ML] −2 mm, ventral 2 mm). A reference electrode was implanted posterior to lambda at a depth of 3 mm. These electrodes were attached to the skull with dental cement. Accuracy of electrode placement was later confirmed using histology. An electromyography (EMG) electrode and reference were sutured to the nuchal muscles, and the telemetry package implanted subcutaneously under the dorsal skin overlying the animal’s back. Immediately following surgery, animals were administered subcutaneous buprenorphine (0.03 mg/kg) for pain management and allowed to recover in the home cage (3 days for weanling aged pups, 1 week for adults), which was placed in individual attenuation chambers to prepare for undisturbed data collection.

**Figure 1:**
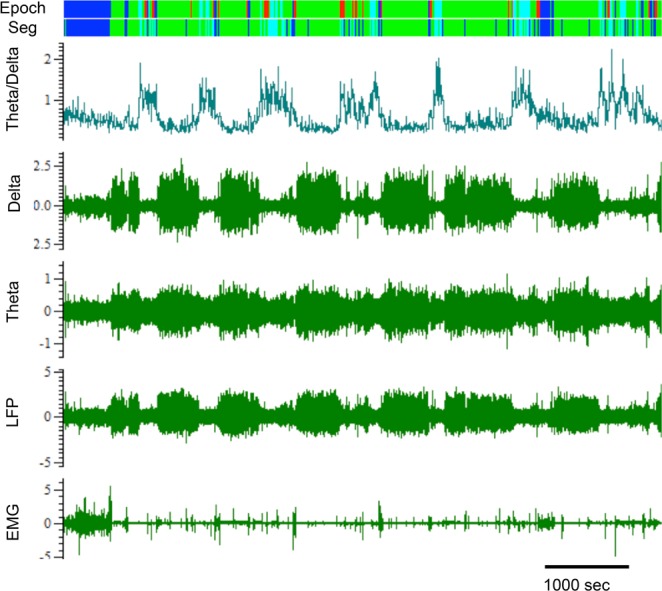
Representative example of sleep staging in a young adult using Spike2’s Rat Sleep Auto scoring algorithm^42^. The record is first scored in 5 second segments (Seg channel) then categorized into 20 second epochs according to the predominant state (Epoch channel). Blue bars indicate periods of wake, green bars indicate periods of NREM sleep, and cyan indicates REM sleep. In rodents, NREM sleep is synonymous with slow-wave sleep (SWS), equivalent to human NREM stages 3 and 4. Red bars indicate uncategorizable epochs as determined by the algorithm.

Following recovery, 24-hour recordings were collected from the telemetry system which included LFP, EMG, general locomotor activity, and body temperature from freely-moving animals in the home cage. The unprocessed biopotentials from the LFP and EMG telemetry channels were digitized (EEG: 1 kHz; EMG 0.5 kHz) for collection with Spike2 software (Cambridge Electronic Design). Artifacts were identified and removed using Spike2’s ArtRem script, and recordings were scored for vigilance states and spectral power for each state (~1 Hz bins) using Spike2’s Rat Sleep Auto scoring algorithm^42^ (Fig. 1), As described in detail in Costa-Miserachs *et al*.^42^, this algorithm calculates vigilance states according to individually normalized (based on mean and standard deviation of rectified EMG and LFP theta and delta), variations in spectral properties from each recording/animal. While similar algorithms have been shown to be accurate in rats as young as postnatal day 12^24,43,44^, we further validated our automated sleep scoring algorithm by two methods. Auto-scored records were visually inspected by the lead researcher to confirm auto-scored vigilance states corresponded to periods with the appropriate spectral properties (e.g. high delta power for epochs scored as NREM, absent muscle tone and high theta:delta ratio for REM); accuracy of scoring was also confirmed via simultaneous video recording over 1–2 hour periods (n = 4) to ensure scored sleep epochs corresponded to behavioral sleep periods. Measures of vigilance states included wake, NREM, and REM bout duration, percent time in each state, and sleep spindle density. The amplitude of cortical SWA was assessed with fast-fourier transform (FFT) analyses using ~1 Hz bins. Vigilance states were analyzed from both the diurnal dark phase and light phase to characterize sleep physiology during animals’ major periods of activity vs. inactivity and rest. Sleep measures were averaged over 2–3 days to reduce potential daily variability; any recording days containing greater than 10% unscorable epochs were excluded from analysis. There was no significant difference in percent of unscorable epochs between Control = (mean = 6.511) and LS rats (mean = 6.576; t(61) = 0.1503, P = 0.899). In 5 cases where unscorable epochs were greater than 10% for all days of recording, animals were excluded from analysis entirely; an additional 10 animals were excluded prior to sleep scoring analysis due to major disruption by recording artifacts or issues with the telemetry system. Spindles were quantified using the same methods described in^45,46^, in which the LFP signal was band-pass filtered at 12–15 Hz, rectified, and smoothed with a time constant of 0.1 s. Events > 2 SD above the mean RMS amplitude, calculated individually for each recording, were automatically detected as spindles (Fig. 6), and spindle density (spindles/minute) was calculated from 20 NREM bouts distributed across a 24-hour period. Spindle analyses were not obtained from 7 animals due to variation in electrode placement and/or recording quality. Diurnal changes in 24-hour locomotor activity levels (quantified in “units” proprietary to the DSI telemetry system) in the home cage were collected via an additional telemetry channel. Daily averages in activity levels were calculated by averaging total hourly activity across at least two complete 24-hour periods of undisturbed recordings (e.g. 9AM on day 1 to 8:59 AM on day 3). Hourly activity counts were calculated by separately averaging activity levels for individual hour bins across at least two days (e.g. mean of activity levels at 9AM from days 1–3, mean of activity levels at 10 AM days 1–3, etc.). Telemetry equipment malfunction resulted in the loss of locomotor activity data from 10 animals. In addition to the home cage activity, open field activity was also assessed in a different group of LS and Control animals at weaning and young adulthood in a novel black Plexiglas arena (45.5 × 30.5 × 45 cm) during a 5 min period.

### Statistical methods

Statistical analyses were conducted using GraphPad Prism version 6.07 software. 2 × 3 ANOVAs were selected based on the experimental design and normality of the data; Sidak post-hoc tests (α = 0.05) were selected to appropriately correct for multiple comparisons while maximizing statistical power for our sample size. Independent samples t-tests were used for LS-CON comparisons of percent time and bout duration, for each age and vigilance state, correcting for multiple comparisons using the Holm-Sidak method. Three separate 2 × 16 (Group × Frequency) repeated measures ANOVAs (RM on Frequency) were used to compare NREM spectral power distributions between CON and LS groups at weaning, young adulthood, and older adulthood, respectively.

Simple mediation analyses were performed on the role of sleep in early life treatment effects on home cage hyperactivity using multivariate regression in SPSS as described in^47^. Combined data across all age groups were used for these analyses. Briefly, linear regression was used to separately determine the relationship between the early life treatment (control = 0 or LS = 1) on the behavioral outcome (home cage hyperactivity) and the early life treatment on the selected sleep parameter. This was followed by multiple regression of the effects of the early life treatment and the potential sleep mediator on home cage hyperactivity. Regression beta values and significance probabilities are reported.

## Results

### Scarcity-adversity model of low shaving (LS) induced maltreatment

To verify that the low-shaving treatment modified maternal behavior towards the pups as we have previously reported^18,19,48^, observations were made on a small sample of pups in LS and control conditions (n = 3/condition, 1 pup/litter). As shown in Table 1, LS treatment induces abusive maternal behavior during mother-pup interactions in the nest, including rough handling of pups, stepping on and dragging of the pups as well as scattered litters, although pups gain weight normally. These aberrant maternal behaviors are typically limited to times when the mother enters and leaves the nest, as she roughly moves her pups within the nest and grooms them, meanwhile stepping on other pups and picking them up from areas other than the species typical nape of the neck. The mother is also more likely to take care of herself, with more eating, drinking and self-grooming while in the nest. While the LS mother takes longer to settle down to nursing her pups, the overall activity level is significantly higher than controls within the nest^49^. Once the mother settles down to nurse, maternal behavior between the control and LS mothers is not statistically different. Pups maintain their attachment to the mother despite this rough handling and continue to seek proximity to the mother and nurse at levels similar to controls.

### Actigraphy: infant LS treatment modified locomotor activity, but only in home cage

**Figure 2:**
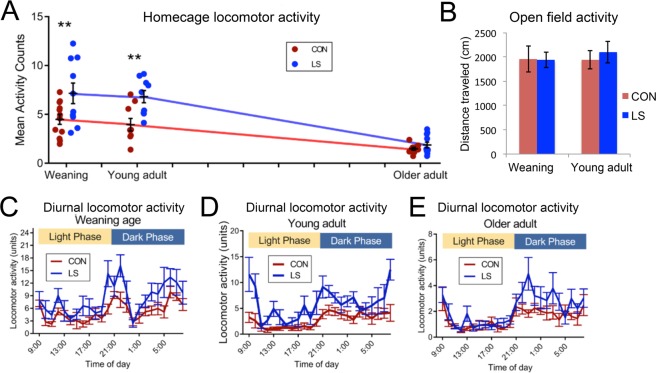
Average daily locomotor activity in the home cage was calculated across at least two days of recording, ensuring there were no disturbances to the animals during this period. Locomotor activity is quantified in “units” proprietary to the DSI telemetry system. (**A**) X-axis ticks represent approximate months of age. LS animals were significantly hyperactive compared to controls (2 way ANOVA, Group × Age, main effect of group, F (1, 47) = 15.56, P = 0.0003). Older animals tended to be less active than younger animals (main effect of age, F (2, 47) = 27.36, p < 0.0001) though the interaction was just short of significance (F (2, 47) = 3.014, P = 0.0586). Sidak multiple comparisons tests (P < 0.05) revealed significant post-hoc differences between controls and LS at weaning (n = 12 controls and n = 10 LS) and intermediate age (n = 5 controls and n = 5 LS), but not older adulthood (n = 10 controls and n = 11 LS). LS animals were approximately 59% and 123% more active than controls at weaning age and intermediate adulthood, respectively. (**B**) In contrast to the LS home cage hyperactivity in younger animals, there was no significant difference in activity in an open field test between LS and CON animals at either weaning or young adult (t-tests, N.S.). (**C–E**) Detailed analyses of hourly activity levels at each age. The diurnal rhythmicity of locomotor activity levels was normal in LS animals at weaning and older adulthood (Three 2 × 24 RM ANOVAs, non-significant Group × Time of day interactions). However, there was a significant Group × Time of day interaction for animals in the young adult cohort (F (23, 92) = 2.290, P = 0.0029). As expected, there was a significant main effect of Group on diurnal activity levels for weaning (F (1, 6) = 7.058, P = 0.0377; n = 12 Control, n = 10 LS) and intermediate age cohorts (F (1, 4) = 18.33, P = 0.0128; n = 5 controls, n = 5 LS), but not the older adults (n = 10 controls, n = 11 LS).

LS treatment has a number of neurobehavioral impacts that emerge at weaning, some of which have a nonlinear developmental expression, including limbic system dysfunction, depressive-like behavior, altered threat assessment, modified fear conditioning, anxiety, and social deficits,^18–22,34,36,49^. The present experiments extended this behavioral assessment to include levels of locomotor activity in both a novel open field and in the familiar home cage activity using the movement sensor in the telemetry transmitter. These two different activity assays have been shown to be differentially sensitive to genetic background^50^ and pharmacological interventions^50,51^, with some evidence that selective home cage hyperactivity may be a good model for hyperactivity in AD/HD^50,52^. Early-life Scarcity-Adversity LS treatment resulted in later life behavioral hyperactivity in the home cage across development, including at weaning, young adulthood, but not older adulthood (Fig. 2A, 2 way ANOVA of average activity levels, Rearing Group × Age, main effect of group, F (1, 47) = 15.56, P = 0.0003). Older animals were less active than younger animals (main effect of age, F (2, 47) = 27.36, P < 0.0001) though the interaction was just short of significance (F (2, 47) = 3.014, P = 0.0586). Sidak multiple comparisons tests (P < 0.05) revealed significant post-hoc differences between controls and LS at weaning (n = 12 controls, n = 10 LS) and intermediate age (n = 5 controls, n = 5 LS), but not in older adulthood (n = 10 controls, n = 11 LS). On average, home cage activity levels in LS animals were approximately 59% and 123% greater than controls at weaning age and intermediate adulthood, respectively. Detailed analyses of hourly fluctuations in activity levels sampled across the 24 hour light-dark cycle were conducted for each age group (Fig. 2C–E). These indicated that the diurnal rhythmicity of activity levels was normal in LS animals at weaning and older adulthood (three 2 × 24 repeated measures ANOVAs, non-significant Group × Time of day interactions) (though see^53^). However, there was a significant Group × Time of day interaction for animals in the intermediate adult cohort (F (23, 92) = 2.290, P = 0.0029). As expected, there was a significant main effect of Group on diurnal activity levels for weaning (F (1, 6) = 7.058, P = 0.0377; n = 12 Control, n = 10 LS) and intermediate age cohorts (F (1, 4) = 18.33, P = 0.0128; n = 5 controls, n = 5 LS), but not the older adults (n = 10 controls, n = 11 LS). The lack of an effect in older adults may reflect a floor effect due to cage size and age-related sedentariness.

Using data from a separate cohort of LS and control animals (to avoid additional handling which could confound our 24 hr sleep measures), we showed that the hyperactivity did not extend to a non-familiar, open field testing apparatus. No differences in activity were detected in an open field (Fig. 2B) in either weaning (Control, n = 10, LS, n = 14, t(22) = 0.32, p = 0.754) or young adults (Control, n = 15, LS, n = 15, t(28) = 0.55, p = 0.587), suggesting context is a critical variable for expression of hyperactivity. Time spent in center vs. walls of OFT was analyzed in the same animals and no differences were observed between groups at either age. Percent time in center, PN60: Control 32.74 ± 2.75; LS 34.42 ± 3.53, p = 0.713; PN23: Control 58.14 ± 1.06, LS 57.81 ± 0.78.

### Sleep architecture: early life trauma impacts older adults

**Figure 3:**
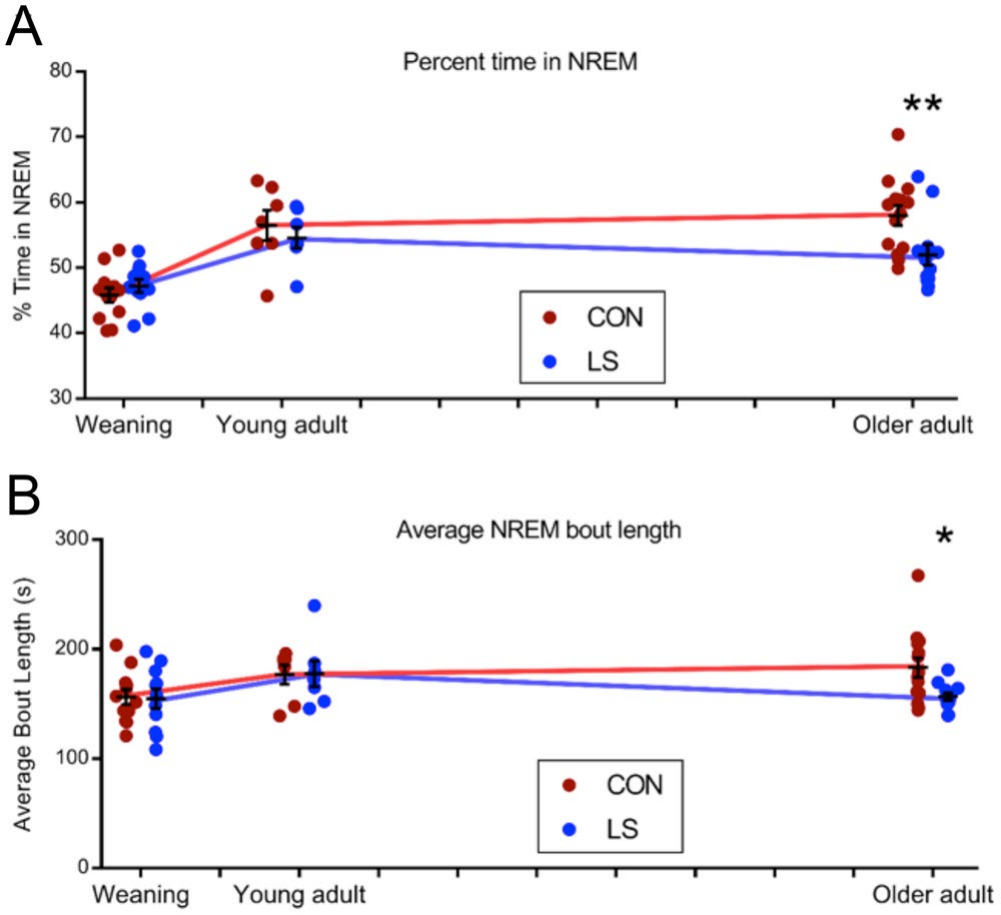
The developmental trajectories of NREM sleep parameters differed between LS and control animals. For each analysis, n = 12 Control, n = 11 LS at weaning age; n = 7 Control, n = 7 LS young adults; n = 14 Control, n = 12 LS older adults. (**A**) Percent time in NREM sleep. Deficits in NREM sleep time achieved during the light phase did not appear in LS animals until they reached older adulthood (2 way ANOVA, Group × Age, main effect of age, F (2, 57) = 22.99, P < 0.0001). The main effect of group did not achieve significance (F (1, 57) = 2.849, P = 0.0969), though there was a significant Group × Age interaction (F (2, 57) = 3.541, P = 0.0355). Sidak multiple comparison tests revealed a post-hoc difference only in older adults. **P < 0.01. X-axis ticks represent approximate months of age. (**B**) Average NREM bout length. The duration of each bout of NREM sleep was averaged across the light phase. A similar pattern emerged in that NREM sleep fragmentation was not evident in LS animals until older adulthood (2 way ANOVA, main effect of age, F (2, 57) = 3.335, P = 0.0427). Sidak multiple comparison tests revealed a post-hoc difference only in older adults. *P < 0.05. X-axis ticks represent approximate months of age.

Examination of vigilance states across all ages in the two groups revealed that early-life Scarcity-Adversity LS treatment in infancy substantially impacted sleep, with distinct age-specific effects most pronounced in older adults and appeared selective to NREM. Specifically, while the duration of NREM sleep was not affected by LS treatment in weaning and younger adult animals, older adult LS animals showed decreased NREM sleep time during the light phase compared to controls (Fig. 3, 2 way ANOVA, Group × Age, main effect of age, F (2, 57) = 22.99, P < 0.0001; n = 23 weaning, n = 14 young adult, n = 26 older adult). There was a significant Group × Age interaction (F (2, 57) = 3.541, P = 0.0355) and no main effect of group (F (1, 57) = 2.849, P = 0.0969). Sidak multiple comparison tests revealed a post-hoc difference only in older adults (P = 0.0085; n = 14 controls, n = 12 LS), in which LS animals spent less time in NREM (M = 51.99) than controls (M = 57.96) during the light phase. LS-related effects on percent time in NREM were not detected in the dark phase; a Group × Age 2 way ANOVA of percent time in NREM sleep achieved during the dark phase revealed only a significant main effect of age (F (2, 57) = 15.25, P < 0.0001), but no effect of group nor Group × Age interaction (data not shown). Significant age-dependent effects of LS were not detected in REM sleep during either the light or dark phase.

In addition to the decreased duration of NREM sleep, NREM was significantly more fragmented in older LS animals compared to controls. NREM sleep was more fragmented later in the lifespan (2 way ANOVA of NREM bout length, main effect of age, F (2, 57) = 3.335, P = 0.0427). Sidak multiple comparison tests revealed LS animals experienced a reduction in light phase NREM bout length only in older adulthood (M = 156.8 vs 183.3 for controls, P = 0.0393). In contrast to changes in NREM sleep time, which were only detected during the light phase when rats achieve the bulk of their sleep, age-dependent effects of LS on NREM sleep fragmentation persisted into the dark phase (2 way ANOVA of NREM bout length, Group × Age, main effect of age, F (2, 57) = 4.643, P = 0.0134; significant Group × Age interaction, F (2, 57) = 4.221, P = 0.0195). Sidak post hoc tests revealed that differences were only detected in older adulthood: LS rats’ dark phase NREM sleep was fragmented into shorter bouts (M = 128.2 seconds) than controls (M = 153.8 seconds, P = 0.036, data not shown).

**Figure 4:**
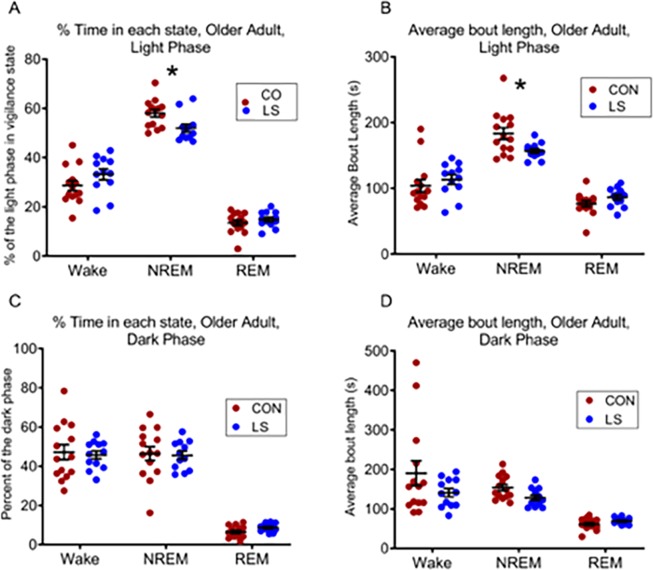
Late-life changes in sleep/wake architecture due to LS treatment appear constrained to NREM. (**A–C**) Comparing the percent time of the light phase (LP) and dark phase (DP) spent in each vigilance state (Wake, NREM, and REM) revealed a difference only in NREM, specifically, during the light phase (**B**, LS animals spent a less time in NREM sleep (M = 51.99%) than controls (M = 57.96%; independent samples t-test, *t*(24) = 2.73, P = 0.023). (**D–F**) The length of each bout of Wake and REM did not differ between aged LS and control animals. However, LS animals had significantly shorter NREM bouts in both the light phase (M = 183.3 for LS, vs. M = 156.8 for controls; independent samples t-test, *t*(24) = 2.62, P = 0.029) and the dark phase (M = 153.8 for LS, M = 128.2 controls; independent samples t-test, *t*(24) = 2.403, p = 0.029). For each analysis, n = 12 Control, n = 11 LS at weaning age; n = 7 Control, n = 7 LS young adults; n = 14 Control, n = 12 LS older adults. *LP* = *light phase; DP* = *dark phase*, *P < *0.05*.

Late-life changes in sleep/wake architecture due to LS treatment appear constrained to NREM. (Fig. 4.) Separate independent sample t-tests were conducted for each vigilance state (Wake, NREM, and REM) comparing the percent time spent in state between LS and controls, at each developmental time point. As expected, older adult LS animals spent less of the light phase in NREM sleep (M = 51.99%) than older adult controls (M = 57.96%; independent samples t-test, *t*(24) = 2.73, P = 0.023).

The length of each bout of Wake and REM did not differ between aged LS and control animals. However, LS animals had significantly shorter NREM bouts in both the light phase (M = 183.3 for LS, vs. M = 156.8 for controls; independent samples t-test, *t*(24) = 2.62, P = 0.015) and the dark phase (M = 153.8 for LS, M = 128.2 controls; independent samples t-test, *t*(24) = 2.403, p = 0.024). For each analysis, n = 12 Control, n = 11 LS at weaning age; n = 7 Control, n = 7 LS young adults; n = 14 Control, n = 12 LS older adults. The percent time in each vigilance state (NREM, REM, and Wake), and the average bout length for each state, were separately analyzed in older adults (n = 14 Controls and n = 12 LS). Sleep/wake architecture did not appear to be impacted by LS rearing in weaning age or young adult animals, in either the light or dark phase (statistics summarized in Tables 2 and 3).

**Table 2:** Comparisons of percent time in each vigilance state (wake, NREM, and REM) between LS and control groups are summarized at three points across development.

Age	Phase	*n*	Group	W			N			R		
				*M*	*SD*	*P*	*M*	*SD*	*P*	*M*	*SD*	*P*
Older Adult	LP	12	CON	28.60	7.58	*NS*	57.96	5.62	0.023	13.44	4.21	*NS*
			LS	33.18	7.64		51.99	5.52		14.84	3.20	
	DP	11	CON	47.14	14.39	*NS*	46.40	13.04	*NS*	6.46	3.00	*NS*
			LS	45.76	7.07		45.58	7.27		8.66	2.02	
Young Adult	LP	7	CON	27.12	6.72	*NS*	56.48	6.08	*NS*	16.40	3.78	*NS*
			LS	28.93	4.25		54.58	4.26		16.50	3.17	
	DP	7	CON	58.30	6.97	*NS*	36.45	6.37	*NS*	5.24	1.81	*NS*
			LS	60.88	8.42		33.90	6.98		5.21	2.10	
Weaning	LP	14	CON	39.69	4.83	*NS*	45.81	3.85	*NS*	14.50	2.86	*NS*
			LS	37.36	4.29		47.24	3.38		15.40	3.74	
	DP	12	CON	59.60	8.81	*NS*	31.39	6.76	*NS*	9.02	2.76	*NS*
			LS	60.18	8.45		31.16	6.63		8.66	2.48	

T-tests conducted on binned data were corrected for multiple comparisons using the Holm-Sidak method; multiplicity-adjusted P values are reported. Significant differences (*P < 0.05) were reported; all other effects were non-significant.

**Table 3:** Comparisons of average bout length in each vigilance state (wake, NREM, and REM) between LS and control groups are summarized at three points across development.

Age	Phase	*n*	Group	W			N			R		
				*M*	*SD*	*P*	*M*	*SD*	*P*	*M*	*SD*	*P*
Older Adult	LP	12	CON	103.87	35.54	*NS*	183.34	33.23	0.03	76.67	17.08	*NS*
			LS	113.34	25.45		156.80	11.89		86.35	14.38	
	DP	11	CON	190.16	117.81	*NS*	153.82	30.11	0.03	61.43	14.10	*NS*
			LS	141.00	36.37		69.47	8.10		8.10	69.47	
Young Adult	LP	7	CON	111.34	47.01	*NS*	176.82	23.22	*NS*	93.55	17.57	*NS*
			LS	130.11	41.23		177.54	31.10		95.52	17.67	
	DP	7	CON	207.18	78.83	*NS*	117.77	33.79	*NS*	76.84	28.39	*NS*
			LS	261.04	80.91		121.36	16.22		72.07	12.95	
Weaning	LP	14	CON	170.00	41.83	*NS*	156.41	23.72	*NS*	85.60	18.89	*NS*
			LS	370.84	209.80		138.68	20.61		83.71	15.17	
	DP	12	CON	147.29	44.77	*NS*	154.81	29.26	*NS*	93.23	21.47	*NS*
			LS	379.56	129.42		151.75	23.74		89.50	19.73	

T-tests conducted on binned data were corrected for multiple comparisons using the Holm-Sidak method; multiplicity-adjusted P values are reported. Significant differences (*P < 0.05) were reported; all other effects were non-significant.

### Sleep neurophysiology: early life trauma impacts young adults

**Figure 5:**
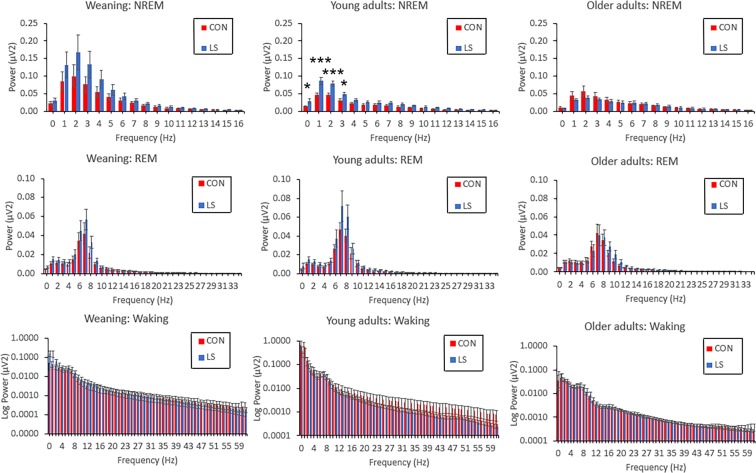
LFP spectral power (mean ± SEM) of in each vigilance state in each group. Using the Spike2 Rat Sleep Auto script, Fourier analyses using approximately 1 Hz bins were performed on each animal’s LFP signal across all epochs detected for each state in a 24-hour period. The results were compared between CON and LS animals using separate Group × Frequency RM ANOVAS for each age group and state. Plots of FFT power results were adjusted to maximize visualization of relevant frequency bands in each state, and the data were plotted as log power for waking to visualize higher frequencies All age groups in each state had a significant main effect of frequency., Only the Young Adult group during NREM state showed a significant main effect of Group and significant Group × Frequency interaction. Post-hoc tests revealed that LS animals’ spectral power was significantly elevated in the lower delta frequency bands (0–3 Hz). *P < 0.05; ***P < 0.0001. No other age or vigilance state showed a main effect of group. In young adults there was a significant group × frequency interaction during REM (F (35, 420) = 1.774, P = 0.0051),and in weanings there was a significant interaction in waking FFT power (F (102, 1530) = 4.493, P < 0.0001), though post-hoc tests revealed no significant pairwise differences in either case. (n = 11 control, n = 10 LS weaning; n = 7 control, n = 7 LS young adults, n = 10 control, n = 9 LS older adults).

While sleep architecture was not modified by LS treatment until the animals were aged, other aspects of sleep physiology were affected at earlier ages. For example, LS treatment produced enhanced SWA power in young animals compared to controls, though this enhancement only reached significance in the young adult age group. An analysis of FFT for each age revealed that all age groups had a significant main effect of frequency (3 separate 2 way repeated measures ANOVAs, Group × Frequency, Fig. 5A–C), but only the Young Adult cohort (Fig. 5B; n = 7 in each group) showed a significant main effect of Group (F(1, 12) = 9.25, P = 0.0102) and significant Group × Frequency interaction (F(16, 192) = 7.801, P < 0.0001). Post-hoc tests revealed that young adult LS animals’ spectral power was significantly elevated in the low delta band (0–3 Hz) bins.

In contrast to the effects on SWA power during NREM sleep, there were no significant effects of early life trauma on FFT analyses during other vigilance states at any age (group × frequency ANOVA’s). During REM sleep, FFT’s (0–35 Hz) showed main effects of frequency at all ages (weaning, F (1, 35) = 35.14, P < 0.0001; young adult, F (1, 35) = 46.90, P < 0.0001; older adult, F (1, 35) = 35.03, P < 0.0001) but no significant main effect of group. In young adults there was a significant group × frequency interaction during REM (F (35, 420) = 1.774, P = 0.0051), though post-hoc tests revealed no significant pairwise differences. Similarly, during waking, there were no significant main effects of group in FFT power analyses (0–100 Hz to include gamma band activity during waking), though there were significant main effects of frequency at each age (weaning, F (1, 102) = 14.91, P = 0.0010; young adult, F (1, 102) = 6.79, P = 0.021; older adult, F (1,102) = 12.27, P = 0.0016). There was significant group × frequency interactions in waking FFT power in weaning animals (F (102, 1530) = 4.493, P < 0.0001) though post-hoc tests revealed no significant pairwise differences at any frequency.

**Figure 6:**
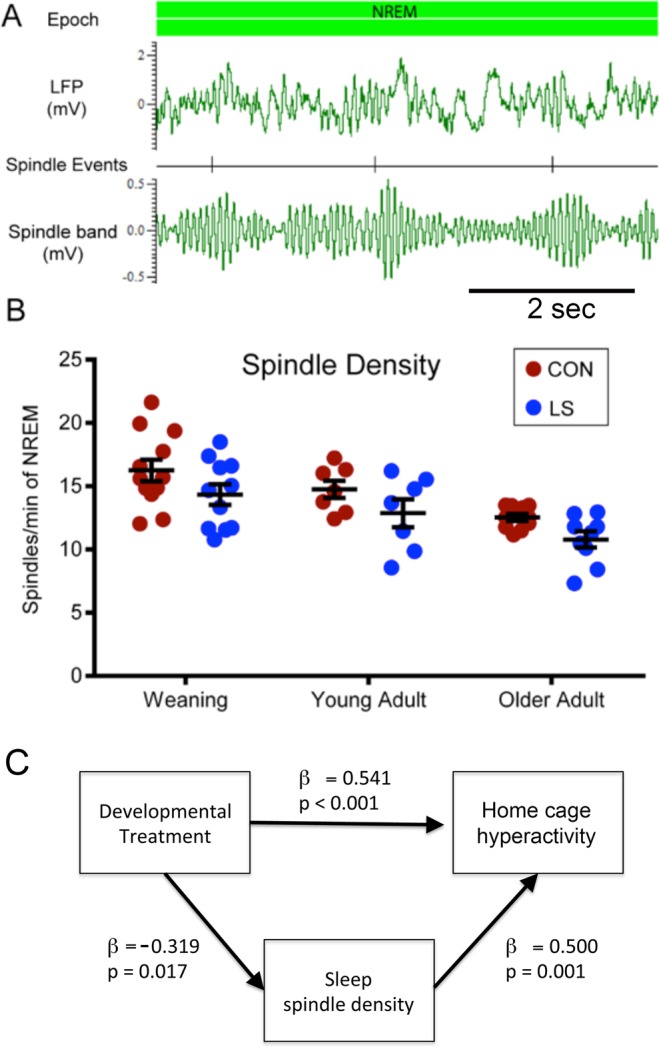
Sleep spindle detection and analysis. (**A**) Representative example of sleep spindle detection procedure. Spindles were quantified during NREM periods by filtering cortical LFP between 12–15 Hz. Periods during which spindle band RMS amplitude exceeded the mean by 2 standard deviations were marked as spindle events. (**B**) NREM sleep spindle density across the lifespan. There was a main effect of LS treatment on spindle density (F (1, 50) = 8.283, P = 0.0059) and a main effect of Age (F (2, 50) = 12.46, P < 0.0001), though there was no significant interaction. Post-hoc analyses indicated that LS-induced reductions of approximately 12% at weaning (n = 12 Control, n = 11 LS), 13% in young adulthood (n = 7 control, n = 7 LS), and 14% in older adulthood (n = 10 Control, n = 9 LS) did not achieve statistical significance. (**C**) Mediation analysis of the relationship between early life treatment (control or LS), sleep spindle density and home cage hyperactivity. Sleep spindle density significantly mediated the effects of early life treatment on subsequent behavioral hyperactivity. See text for additional details.

One aspect of sleep that was affected across all age groups by LS treatment was an overall reduction in NREM sleep spindle density (Fig. 6, 2 way ANOVA, Group × Age, significant main effect of Group, F (1, 50) = 8.283, P = 0.0059). Spindle density also decreased with age, even in control animals (main effect of Age, F (2, 50) = 12.46, P < 0.0001). There was no significant Group × Age interaction. Although there was a main effect of group in the ANOVA, post-hoc analyses indicated that LS-induced reductions of approximately 12% at weaning (n = 12 Control, n = 11 LS), 13% in young adulthood (n = 7 control, n = 7 LS), and 14% in older adulthood (n = 10 Control, n = 9 LS) did not achieve statistical significance.

As noted above, our sleep monitoring system also included measures of general activity level within the home cage, which showed a consistent phenotype of home cage hyperactivity. In order to determine the relationship between early life treatment, sleep and later-life behavioral outcomes, we used multiple regression techniques to perform an analysis of whether sleep significantly mediated the effects early life LS rearing on later life home cage hyperactivity. As shown in Fig. 6C, sleep spindle density significantly mediated the effects of LS treatment on behavioral hyperactivity in the home cage. No other sleep measure showed a significant mediation effect (not shown).

## Discussion

Early-life maltreatment, as induced by the Scarcity-Adversity model of LS, produced sleep dysfunction, which was expressed in the youngest pups and continued into adulthood. Specifically, early life maltreatment produced a long-lasting constellation of alterations in sleep, which has distinct developmental trajectories: minor disturbances in sleep architecture at weaning, such as NREM sleep spindle density, that progressed to include SWA amplitude in young adults, and further progressed in older adults as fragmented NREM sleep and functional insomnia. Since early life trauma-induced behavioral dysfunction is expressed in infancy^18,20,21^, yet insults to NREM integrity only appear by later life, it is unlikely that macro-structural sleep deficits are an early cause or contributing factor towards the development of juvenile-onset cognitive/behavioral disturbances due to early trauma. However, the reduction in sleep spindle density, which emerges early in life, was a significant mediator of the effects of early trauma on home cage hyperactivity. Thus, the results suggest that early developmental events may be an important contributor to variability in sleep health in aging. Sleep disruption is an important risk factor for aging related disorders^1^ and dementia-related beta-amyloid accumulation^54^.

A careful analysis of sleep neurophysiology reveals a complex picture of trauma-induced sleep deficits across development. Here we provide the first evidence that early maltreatment affects not only sleep architecture, but also SWA amplitude and NREM sleep spindle density. Importantly, these latter effects are present across the entire lifespan beginning by at least weaning. For example, sleep spindle density shows a reduction of over 10% at each age tested here. Sleep spindles are critical for memory consolidation^55^ and emotional regulation^14,15,56^. Thus, sleep spindles present a possible target for therapeutic intervention in this population. Spindle enhancement via pharmacology, and more experimentally, transcranial stimulation, appears to improve memory in healthy individuals and reduce cognitive deficits in patients with schizophrenia^56^.

The onset and maintenance of sleep relies on a balance between two opposing systems: one promoting sleep, and the other promoting wakefulness. Insomnia can result from disruption of either system, and thus the mechanisms of the LS-induced sleep disruption observed here is unknown. Within the NREM sleep promoting system, inhibitory GABAergic processes quiet wake promoting regions^57–59^. LS treatment has been shown to modify synaptic inhibition within the amygdala^22^ and parvalbumin expressing GABAergic synaptic structure^49^, though no differences in GABAergic PV cell numbers were observed here in sleep related forebrain structures (Supplementary Fig. 1). Examination of other cell types involved in sleep-wake transitions and sleep maintenance, such as somatostatin^57,60,61^ or neuropeptidergic neurons^62^ is warranted. In addition, there is evidence that early trauma heightens locus coeruleus (LC) function^63^ a brain area strongly linked to sleep/wake cycling^64^, which when combined with our home cage hyperactivity, may suggest neurobehavioral abnormalities in the arousal response. Heightened arousal that persists into sleep is a potential mechanism of the LS-induced NREM disruptions.

We also found that infant trauma-produced sleep disruption included NREM SWA power elevation relative to controls at young adulthood. SWA diminishes in tandem with cortical maturation^11–13^, and therefore, developmentally inappropriate SWA may indicate a maturational delay. In line with the present results of significant increases in 1–3 Hz oscillation power after LS, similar increases in this frequency range have been shown in humans with depression^65^ and ADHD^66^.

Finally, in addition to these abnormalities in SWA, our results provide novel evidence of hyperactivity-like behavioral pathology as a direct result of developmental trauma, a behavior considered by some to be a symptom of ADHD^67^, especially when not expressed in a novel environment and limited to the home cage^68^. Future research should assess whether the sleep abnormalities identified here may be viable biomarkers, or even therapeutic targets, for ADHD-like behavioral pathology.

These sleep disturbances were caused by aberrant maternal behavior associated with rough treatment of pups was induced by the Scarcity Adversity Model of LS. This treatment produces ubiquitous effects on the brain and behavior, some of which are initiated during infancy and impact the amygdala and hippocampus^40^, while other emerge before or after weaning to impact the prefrontal cortex^41,69^. The developmental trajectory of this adversity is not necessarily linear: fear learning is enhanced in both infancy and adulthood but attenuated during adolescence^41^. While each paradigm of early life adversity produces a unique outcome and developmental trajectory, there is a consistent finding of ubiquitous neurobehavioral effects and nonlinear developmental trajectory^48,53,70,71^. Thus, as we integrate the present sleep results into the early-life trauma literature in rodents and attempt to translate across species, we suggest that the disrupted sleep induced by early life trauma introduces a continued adversity that potentially amplifies trauma effects across the life span.

Our study highlights trauma with the caregiver in a rodent model, which complements the myriad other infant experience models. These models range from assessing early life stress experienced by variations of typical maternal care^72^, to models involving adversity, including the maternal separation/deprivation model (prolonged removal of maternal sensory stimulation of pups), exposure to trauma (shock or elevated stress hormones), brief novelty exposure outside the nest, and handling^19–21,28,29,35–37,40,53,71,73–76^. It should be noted that various labs have employed variations of the low bedding paradigm^77^, including a version using a wire-mesh floor, the present model is a milder version in which pups gain weight normally and reach normal developmental milestones^78^. These models converge in identifying the stress system as a mechanism mediating enduring adult outcomes.

In conclusion, our developmental approach has highlighted that early life trauma produces developmentally dynamic changes in sleep disturbances, beginning with alterations in spindles known to be important for brain development in pups, and progresses to later life fragmentation of NREM sleep and insomnia. Sleep deficits can be extremely debilitating, causing dysphoria and daytime impairment at any age. In older adults in particular, sleep fragmentation predicts worse daytime functioning, including cognitive and social impairment^79^. Given the importance of sleep in cognitive and emotional functions, these results highlight an important factor driving variation in sleep and cognition throughout the lifespan that suggest age-appropriate and trauma informed treatment of sleep problems^80–88^.

## Supplementary information

Supplementary Information
