# Supplementary material for: Early Life Trauma Has Lifelong Consequences for Sleep And Behavior
Source: Sci Rep. 2019 Nov 13;9:16701. doi: 10.1038/s41598-019-53241-y (PMC6853921; doi:10.1038/s41598-019-53241-y)
Supplement: Supplementary file 1 — Supplementary Information [file 41598_2019_53241_MOESM1_ESM.pdf]

SUPPLEMENTARY INFORMATION

for

EARLY LIFE TRAUMA HAS LIFELONG CONSEQUENCES FOR SLEEP AND BEHAVIOR

Monica Lewin[1,2], Jenna Lopachin[1], James Delorme[1,4], Maya Opendak[1,3], Regina M. Sullivan[1,3,5] and Donald A. Wilson[1,3,5]

[1]Nathan Kline Institute for Psychiatric Research, Orangeburg, NY

[2]Sackler Neuroscience Graduate Program, NYU School of Medicine, New York, NY

[3]Department of Child and Adolescent Psychiatry, NYU School of Medicine, New York, NY

[4]Neuroscience Graduate Program, University of Michigan, Ann Arbor, MI

[5]*Joint senior authors*

Corresponding author: Donald Wilson, Nathan Kline Institute, 140 Old Orangeburg Road, Orangeburg, NY 10962, 845-398-2178, Donald.wilson@nyumc.org

Short/running title: Early life trauma, sleep and behavior

Keywords: early-life trauma, sleep, slow-wave sleep, hyperactivity, sleep spindles, sleep in aging, adversity rearing

SUPPLEMENTARY INFORMATION

The control of sleep and sleep-related processes is mediated and modulated by a wide range of cell types and peptides, though sleep is heavily dependent on GABAergic modulation [1,2]. GABAergic projections from sleep-promoting nuclei, such as those found in the basal forebrain (BF), are required for sleep-wake regulation [3,4]. Cortical slow-wave activity is GABA-dependent [5], and sleep-related oscillations such as sleep spindles rely on GABAergic parvalbumin (PV) cell populations in the reticular nucleus of the thalamus (RT) [6]. There is also abundant evidence for GABAergic dysfunction in psychiatric disease [7,8]. GABAergic neurons are sensitive to developmental events, for example following early life adversity [9] and developmental alcohol exposure [10-12]. There is also a strong link between infant trauma-induced psychopathology and GABAergic function in both the human and animal literature [9,13]. For example, recent work from our group demonstrated that early life adversity, using the same Scarcity-Adversity model, reduces output synapses from GABAergic parvalbumin (PV) neurons in the amygdala [9]. Similarly, the more severe treatment of developmental ethanol exposure results in a variety of later life behavioral abnormalities and functional insomnia, as well as decreases in cortical and hippocampal PV neurons [10-12]. Thus, we hypothesized that any sleep disturbances resulting from early trauma may similarly be associated with PV cell loss in sleep-related areas.

Among other populations of neurons, PV expressing GABAergic interneurons are involved in sleep-wake transitions (basal forebrain PV neurons, [4]), sleep spindle activity (reticular thalamic PV neurons; [14]) and cortical SWA (cortical PV neurons; [5]). In a subset of the oldest aged animals examined here, PV+ cell density was determined in basal forebrain, reticular thalamus, somatosensory cortex and piriform cortex as representative cortical and non-cortical regions relevant for sleep-related activity.

Supplementary Methods: Immunohistochemistry and cell counting. At the end of recordings, rats were deeply anaesthetized with urethane before perfusion-fixation with phosphate-buffered saline (PBS) followed by 4% paraformaldehyde. Brains were extracted then immersed in 4% paraformaldehyde for 24 hours, then transferred to 20% glycerol in 0.1% sodium azide cryoprotective solution stored at 4^{0} C for 2-5 days before being cut into 40µm coronal sections. Parvalbumin immunohistochemistry was conducted using Vectafluor R.T.U. Antibody kit with rabbit anti-Parvalbumin (PV27, Swant, Marly, Switzerland). Stained slides were mounted with fluoromount. LS and control age- and sex-matched pairs were processed simultaneously to avoid differences in basal staining. Cell densities were calculated in basal forebrain (BF), somatosensory cortex (SC), thalamic reticular nucleus (RT), and piriform cortex (PCX) by manual cell counts normalized to the area (mm^2) in which counting was performed, which was optimized to circumscribe each structure. Cell densities for each region were averaged bilaterally across at least 3 sections/region/animal. 2 animals were lost in the analysis of BF staining and 3 lost from the analysis of RT staining due to damage to tissues during staining.To assay for potential differences in PV expression, fluorescence intensities of PV+ cells were quantified in reticular thalamus, selected for its high PV cell composition and apparent trend towards an effect of group on cell density. A 2 X 4 (Group X Area) repeated measures ANOVA was used to test for differences in PV cell density in older adult CON and LS rats; the use of repeated measures across both factors was intended, along with our paired design, to control for potential batch differences in immunostaining, and was justified by a significant effect of subject pairing. Similarly, a two-tailed paired t-test (also justified by significant pairing) was conducted to assay for any differences in fluorescence intensity due to levels of PV expression.

Supplementary Results: Parvalbumin Immunohistochemistry: Early life trauma does not impact PV cell numbers

Although sleep measures were affected in older adult rats by early life LS treatment, as shown in Supplementary Fig. 1 there was no detectable effect of LS rearing on PV cell densities in the basal forebrain (BF; N = 6 CON, N = 6 LS), somatosensory cortex (SC; N = 8 CON, N = 8 LS), piriform cortex

(PCX; N = 8 CON, N = 8 LS), or thalamic reticular nucleus (RT; N = 5 CON, N= 5 LS) of older adult rats (2 way repeated measures ANOVA, main effect of area, $F(3,23) = 63.74$, $P < 0.0001$; main effect of group was not significant, $F(1,23) = 2.505$, $P = 0.127$), non-significant interaction). To determine whether cell counts could have been influenced by parvalbumin protein expression levels, mean parvalbumin fluorescence (arbitrary units) of reticular thalamic (RT) PV cells were quantified and did not differ significantly between LS (mean = 34.08 ± 3.553) and controls (mean = 35.02 ± 3.593, $t(4) = 0.671$, $P = 0.539$), suggesting LS treatment did not impact later-life parvalbumin expression levels in this region. PV cell density was not examined in earlier aged animals.

Supplementary Figure 1

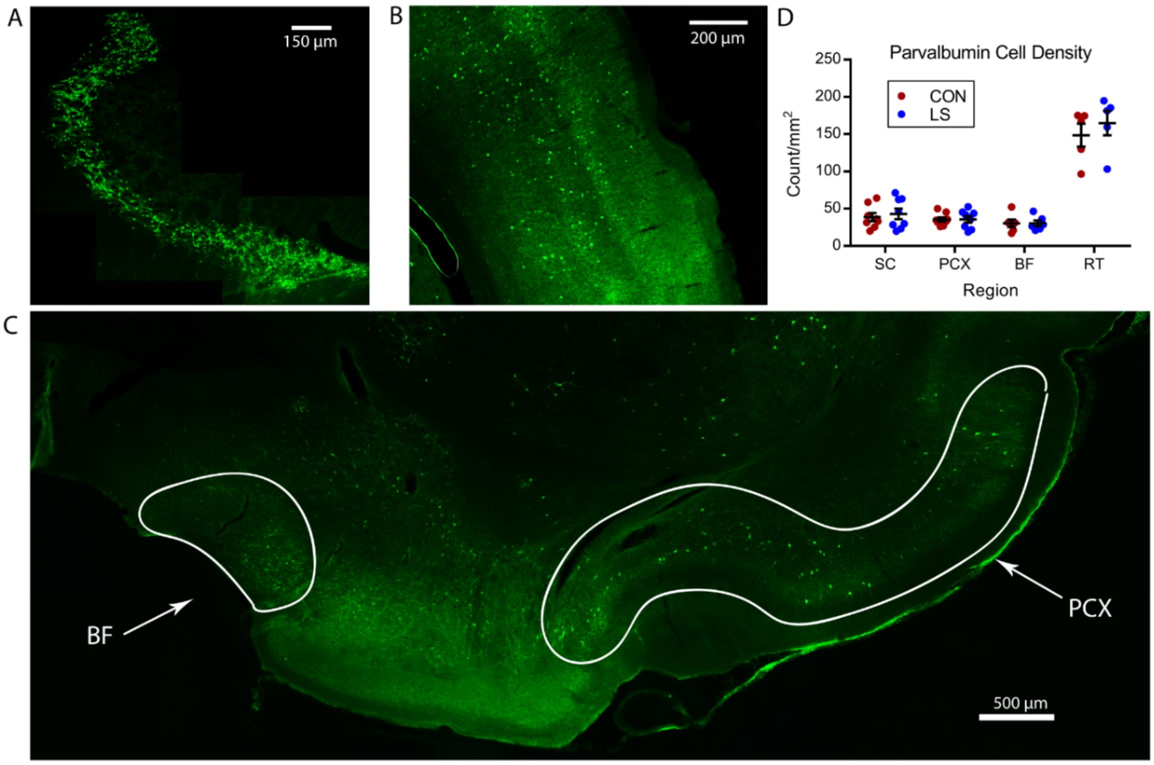

Supplementary Figure 1. Parvalbumin immunohistochemistry and cell density analysis. Parvalbumin (PV) expressing cells were counted in four different regions of older adult LS and Control rats. **A-C.** Representative images of PV expression and counting areas in reticular thalamus (RT; N = 5 CON, N= 5 LS), somatosensory cortex (SC; N = 8 CON, N = 8 LS), basal forebrain (BF; N = 6 CON, N = 6 LS), and piriform cortex (PCX; N = 8 CON, N = 8 LS). **D.** PV+ cell density was calculated (mean cell count/mm^2) for each structure per animal. PV cell density varied by region, but no effect of LS on PV cell density was detected in the selected structures.

1 Luppi, P. H., Peyron, C. & Fort, P. Not a single but multiple populations of GABAergic neurons control sleep. *Sleep Med Rev* **32**, 85-94, doi:10.1016/j.smrv.2016.03.002 (2017).

2 Brown, R. E. & McKenna, J. T. Turning a Negative into a Positive: Ascending GABAergic Control of Cortical Activation and Arousal. *Frontiers in neurology* **6**, 135, doi:10.3389/fneur.2015.00135 (2015).

3 Manfridi, A., Brambilla, D. & Mancia, M. Sleep is differently modulated by basal forebrain GABA(A) and GABA(B) receptors. *American journal of physiology. Regulatory, integrative and comparative physiology* **281**, R170-175 (2001).

4 Xu, M. *et al.* Basal forebrain circuit for sleep-wake control. *Nat Neurosci* **18**, 1641-1647, doi:10.1038/nn.4143 (2015).

5 Steriade, M., Dossi, R. C. & Nunez, A. Network modulation of a slow intrinsic oscillation of cat thalamocortical neurons implicated in sleep delta waves: cortically induced synchronization and brainstem cholinergic suppression. *Journal of Neuroscience* **11**, 3200-3217, doi:10.1523/jneurosci.11-10-03200.1991 (1991).

6 Halassa, M. M. *et al.* Selective optical drive of thalamic reticular nucleus generates thalamic bursts and cortical spindles. *Nature neuroscience* **14**, 1118-1120, doi:10.1038/nn.2880 (2011).

7 Marín, O. Interneuron dysfunction in psychiatric disorders. *Nature Reviews Neuroscience* **13**, 107, doi:10.1038/nrn3155 (2012).

8 Gauthier, I. & Nuss, P. Anxiety disorders and GABA neurotransmission: a disturbance of modulation. *Neuropsychiatric Disease and Treatment* **Volume 11**, 165-175, doi:10.2147/ndt.s58841 (2015).

9 Santiago, A. N., Lim, K. Y., Opendak, M., Sullivan, R. M. & Aoki, C. Early life trauma increases threat response of peri-weaning rats, reduction of axo-somatic synapses formed by parvalbumin cells and perineuronal net in the basolateral nucleus of amygdala. *J Comp Neurol*, doi:10.1002/cne.24522 (2018).

10 Lewin, M. *et al.* Developmental ethanol-induced sleep fragmentation, behavioral hyperactivity, cognitive impairment and parvalbumin cell loss are prevented by lithium co-treatment. *Neuroscience* **369**, 269-277, doi:10.1016/j.neuroscience.2017.11.033 (2018).

11 Saito, M. *et al.* Neonatal Ethanol Disturbs the Normal Maturation of Parvalbumin Interneurons Surrounded by Subsets of Perineuronal Nets in the Cerebral Cortex: Partial Reversal by Lithium. *Cereb Cortex*, doi:10.1093/cercor/bhy034 (2018).

12 Wilson, D. A. *et al.* Developmental ethanol exposure-induced sleep fragmentation predicts adult cognitive impairment. *Neuroscience* **322**, 18-27, doi:10.1016/j.neuroscience.2016.02.020 (2016).

13 Cameron, J. L., Eagleson, K. L., Fox, N. A., Hensch, T. K. & Levitt, P. Social origins of developmental risk for mental and physical illness. *J Neurosci* **37**, 10783-10791, doi:10.1523/JNEUROSCI.1822-17.2017 (2017).

14 Halassa, M. M. *et al.* State-dependent architecture of thalamic reticular subnetworks. *Cell* **158**, 808-821, doi:10.1016/j.cell.2014.06.025 (2014).
